# Determinants of maternal health service utilisation among pregnant teenagers in Delta State, Nigeria

**DOI:** 10.11604/pamj.2020.37.81.16051

**Published:** 2020-09-22

**Authors:** Love Chukwudumebi Mekwunyei, Titilayo Dorothy Odetola

**Affiliations:** 1 Anchor University, Ayobo, Lagos, Lagos State, Nigeria,; 2 Department of Nursing, University of Ibadan, Ibadan, Nigeria

**Keywords:** Utilization, pregnancy, teenagers, availability, accessibility

## Abstract

**Introduction:**

the prevailing high maternal mortality and morbidity rate among pregnant adolescents in Nigeria underscores all efforts said to have been made to tackle maternal deaths among this population. Not much research has been done to ascertain the reasons associated with the poor utilisation of Maternal Health Services (MHS) by pregnant teenagers. This study, therefore, explored the extent and determinants of MHS utilisation among pregnant teenagers in Delta State.

**Methods:**

this study made use of a mixed cross-sectional study design. Multi-stage sampling technique was adopted in selecting eight communities while snowballing was used in identifying pregnant teenagers. A structured interviewer-administered questionnaire was used for the data collection from 212 pregnant teenagers and an interview guide was further used to interview 16 pregnant teenagers randomly selected from the communities. Descriptive and inferential data analyses were done using SPSS version 22. Hypotheses were tested using Chi-square test at P≤0.05 level of significance.

**Results:**

seventy per cent of the participants stated that they utilised MHS by visiting an antenatal care centre (ANC) centre at least once during their pregnancy but only 28.3% had ANC attendance that was appropriate with their gestational age. A grand mean ± SD of 3.4714 showed that there is a high level of perception of stigmatisation among pregnant teenagers. Also, married teenagers [86%] were found to utilise MHS more than their single counterparts [67.1%]. A statistically significant association (Chi-square) was found between utilisation of MHS and maternal education [P=0.024], utilisation of MHS and availability/accessibility of MHS facilities [P=0.002], utilisation of MHS and cost of MHS [P=0.001] and utilisation of MHS and coercion/violence from partner [P=0.000].

**Conclusion:**

the level of utilisation of MHS by pregnant teenagers is low with main determinants of use being stigmatisation of pregnant teenagers, availability of health personnel, accessibility to MHS facilities, permission from significant others and cost of MHS.

## Introduction

Pregnant teenagers constitute a high-risk group often highlighted in public debates [1] and teenage pregnancy is one of the main issues in every health care system since it can have harmful implications on girls´ physical, psychological, economic and social status [2]. It is a worldwide phenomenon affecting both developed and developing countries [3]. Delaying the first pregnancy until a girl is at least 18 years of age helps to ensure safer pregnancy and childbirth. It reduces the risk of her baby being born prematurely and underweight [4]. Teenagers have increased risk for poor maternal and infant outcomes and it is widely assumed that they are less likely than older women to use maternal health services. Nevertheless, the evidence on their use of maternal care services is limited and mixed [5,6]. The risk of dying from pregnancy-related causes is twice as high for women aged 15-19 years and five times higher for girls aged 10-14 years as for women aged 20-29 years [7]. Also, if a mother is under 18 years, her baby´s chance of dying in the first year of life is 60 per cent higher than that of a baby born to a mother older than 19 years [8]. Part of this burden has more to do with poor socio-economic status and lack of ante-natal and obstetric care than physical maturity alone [9]. In the past few decades, the issue of adolescent child-bearing has been increasingly perceived as a critical challenge facing modern society [10]. Despite growing programmatic and research interest in addressing the needs of pregnant women, the particular needs of pregnant adolescents have been poorly served and inadequately documented.

The practice of attending to the needs of this group with specialised services has only recently begun and primarily only in developed countries [10]. Also, one of the major causes of maternal deaths has been reported to be inadequate motherhood services such as antenatal care [11]. Despite the programs embarked on by the Nigerian government to reduce this high rate of maternal mortality, the achievement made so far is low as annual percentage decline in MMR from 1990 to 2008 is 1.5% compared to the targeted 5.5% [12]. Pregnancy among teenagers for some is unplanned while for others, the same cannot be said because such are either in marital unions or stable relationships [13]. Most teenagers with unplanned pregnancies do not seek maternal health care services or do so far into the pregnancy [14]. The results of a study conducted in the Niger delta depict that teenage pregnancy has been on the increase in the south-south. This can be attributed to the fact that young girls in the Niger delta have been lured and deceived to respond to the lust of thousands of oil workers. Many of these girls give birth without attending an antenatal clinic or receiving the help of a professional midwife [15]. The third sustainable development goal, “Achieve gender equality and empower all women and girls", cannot be achieved if Nigeria fails to take prompt action to tackle the causes of maternal deaths as well as other deterrents to the effective utilisation of available maternal health care services.

Even though maternal health care utilisation is essential for further improvement of maternal and child health, little is known about the current magnitude of use (among pregnant teenagers) and factors influencing the use of these services in Delta State as most of the studies are outdated and may not represent the current situation of things. Furthermore, other studies on the use of maternal health care services have largely overlooked ´the pregnant teenager´ or merely grouped them with women of reproductive age not minding their peculiarities of being minors, at risk of social stigmatisation as well as being victims of some of our socio-cultural practices negatively affecting maternal health. Also, this study provides a baseline statistics of the utilisation level of maternal health care services in Delta State, Nigeria, thereby providing information on the extant effect to which there is a diverse gap between what is and what is supposed to be by the year 2030. Hence, quantifying the magnitude of actions needed to achieve the expected goal by 2030. Therefore, this study seeks to provide knowledge on the current magnitude of the utilisation of maternal health service among pregnant teenagers in Delta State. It identifies the pattern of utilisation of maternal health care services among married and unmarried pregnant teenagers, assesses the level of perception of stigmatisation among pregnant teenagers and examines the socio-cultural factors affecting the utilisation of maternal health care services in Delta State, Nigeria.

## Methods

**Research design and population:** the research design for the study is a mixed descriptive cross-sectional design. The researcher, therefore, described, examined and explored maternal health services utilisation of its study population and their determining factors using an in-depth interview method via an interview guide and a researcher-developed questionnaire. The study population for this study was 212 pregnant teenagers who attended ANC in the health centres, TBA´s, mission homes and general hospitals of the selected communities as well as those residing in these communities. The total number of individuals in Delta State was obtained (4,098,291); the percentage of teenagers 14.6% was used to obtain the actual number of teenagers in Delta State (596,635). The male to female sex ratio [0.97 to 1.03] was used to extract the actual number of female teenagers (307,267) and the rate of teenage pregnancy (10%) was used to deduce the estimated number of pregnant teenagers in Delta State [12,16].

**Sampling technique:** a multi-stage sampling technique was used to obtain a representative sample of the communities and health facilities. Stage 1: simple random sampling was done to select a representative sample of eight (8) local government areas (which is 30% of the LGAs in Delta State) from a sampling frame of the 25 local government areas in Delta State. The selected LGAs include Aniocha North, Aniocha South, Ethiope East, Ethiope West, Okpe, Oshimili North, Sapele and Ukwani; stage 2: a sampling frame of all the districts in the selected LGAs was drawn and a representative sample of one ward per LGA was selected using simple random sampling. The selected districts/wards include: Ogwashi-Uku Village, Issele-Azagba, Abraka I, Jesse I, Umunede, Ozoro, Amukpe and Koko. Snowballing sampling technique was also used to identify pregnant teenagers. For the qualitative component of this study, two (2) study participants were randomly selected per community making a total of sixteen [12] study participants which were further interviewed using an interview guide. This helped to explore further factors that determine their utilisation or non-utilisation of maternal health services.

**Sample size determination:** the sample size was calculated using Cronbach´s formula for descriptive study: where n=minimum sample size; Zα=confidence level of 95% (standard value of 1.96); P=0.2 i.e. 21% [17]; q=1-p=1-0.20=0.80; d=desired level of significance or precision 5%=0.05; (3.84*0.21*0.8)/ 0.00025 n=243.2; n=243. For finite population correction (for populations <10,000);

$$n^{i}=\frac{n}{1+\frac{n}{N}}$$

where n=sample size and N=size of population of interest n^i^=(243/(1+243/1491)), n^i^=209. At the end of eight (8) weeks, a total of 212 pregnant teenagers in the eight wards participated in the study.

**Research instrument, data analysis and ethical consideration:** data was collected by interview using an interview guide and a researcher administered questionnaire. Face and content validity of the questionnaire were ensured. The researcher developed questionnaire (based on the objectives of the study and thorough literature search) was given to experts in the field of nursing, senior researchers, reproductive health and monitoring and supervisory team for thorough scrutiny. Each item on the instrument was examined for content, clarity, scope and relevance to the study, that is, its ability to answer the research questions and hypotheses. Also, the reliability of the instrument was established by test-retest method with a correlation co-efficient of 0.81, depicting that the questionnaire was reliable. For the qualitative component of this research, an in-depth interview was carried out by the researcher on sixteen (16) consenting pregnant teenagers using a developed interview guide. The information gotten was recorded on tape and reported thematically. In a bid to determine the extent of utilisation of MHS, those respondents, who indicated that they attended ANC, do so in either a PHC or general hospital and whose number of attendances to ANC was adequate with their gestational age were summed up and rationed against the total number of pregnant teenagers (respondents).

In order to determine the socio-cultural factors affecting the utilisation of MHS, Chi-square analysis was used to determine the association between dependent (MHS utilisation) and independent variables (selected socio-cultural factors affecting its utilisation). Some of the underlying assumptions of Chi-square are each observation is independent of all the others, not more than 20% of the expected counts are less than 5 and all individual expected counts are 1 or higher. In order to assess the level of perception of stigmatisation among pregnant teenagers, a decision rule was used. Mean score of ≥2.5 was considered as high perception of stigmatisation, while a score below 2.5 was regarded as low perception of stigmatisation. For the qualitative aspect, NVivo II analysis was used to analyse the data obtained. Data gotten was reported thematically in groups. Content and narrative analyses were done on the data collected and naturally occurring patterns and themes were identified. Ethical approval was obtained from the Ethical Review Board [ERB] in Delta State Ministry of Health, Nigeria.

**Procedure of data collection:** eight (8) research assistants (some of which were health workers and resided in the study area) were recruited and trained on the modalities for instrument administration and collection. The questionnaires were interviewer-administered. In cases where the prospective respondent and guardian/parent agreed to participate in the study, the respondent was interviewed using the structured questionnaire. Daily checking of filled questionnaires was carried out by the researchers at the end of each field day, to avoid incomplete data collection and to ensure accuracy of data. The in-depth interview lasted for a period of 30 minutes each. Sixteen pregnant teenagers consented to participate in an in-depth interview. One of the respondents was 15 years of age (consent was obtained from her father) while the others were between 18 to 19 years. Research participants were assured of the confidentiality of data collected.

## Results

**Demographic data:** from Table 1, the number of pregnant teenagers increased with their age as most of the respondents were between 18 and 19 years of age. On marital status, 73.1% of the study participants were single while 26.9% were married. 81.1% of study participants were primigravida while 18.9% were multigravida.

**Table 1:** demographic data of research participants

Demographics	Categories	Frequency (F)	Percentage (%)
Age (years)	15	16	7.5
	16	22	10.4
	17	39	18.4
	18	44	20.8
	19	91	42.9
	Total	212	100.0
Marital status	Single	155	73.1
	Married	57	26.9
	Total	212	100.0
Ethnic group	Yoruba	14	6.6
	Igbo	34	16.0
	Hausa	2	.9
	Urhobo	101	47.6
	Ijaw	12	5.7
	Others	49	23.1
	Total	212	100.0
Occupation	Student	85	40.1
	Trader	33	15.6
	Farmer	18	8.5
	Apprentice	59	27.8
	Others	17	8.0
	Total	212	100.0
Gestational age	1-3 months	49	23.1
	4-6 months	84	39.2
	7-9 months	79	37.7
	Total	212	100.0
Previous pregnancy	No	172	81.1
	Yes	40	18.9
	Total	212	100.0
Previous parity	No	180	84.9
	Yes	32	15.1
	Total	212	100.0
Religion	Christianity	181	85.4
	Islam	23	10.8
	Others	8	3.8
	Total	212	100.0

**Extent of utilisation of MHS among pregnant teenagers:** Table 2 reveals that 72.2% attended ANC clinics while 27.8% did not. Of the 72.2% that attended, 49.7% utilised PHC´s, 43.1% utilised the general hospitals while 7.2% utilised private hospitals. Sixty (39.2%) had ANC attendance that was appropriate with their gestational age while 93 (60.8%) did not.

**Table 2:** pregnant teenagers utilisation of maternal health care service

Questions		Categories		Frequency (F)
ANC attendance		No		59
		Yes		153
		Total		212
Place of ANC attendance		Primary health centre		76
		General hospital		66
		Private hospitals		11
		Total		153
ANC attendance appropriate with gestational age		Yes		60
		No		93
		Total		153
MHS services used by research participants	Counseling services	No	166	78.3
		Yes	46	21.7
		Total	212	100.0
	Health Education	No	145	68.3
		Yes	67	31.7
		Total	212	100.0
	Lab Tests	No	145	68.3
		Yes	67	31.7
		Total	212	100.0
	Maternal Immunisation	No	157	74.1
		Yes	55	25.9
		Total	212	100.0
	IPT	No	188	88.7
		Yes	24	11.3
		Total	212	100.0

**Perception of stigmatisation among pregnant teenagers:** from Table 3, mean score of ≥2.5 was considered as high while score below 2.5 was regarded as low. Grand mean ± SD of 3.4714 shows that there is a high perception of both personal and perceived stigmatisation among pregnant teenagers in Delta State.

**Table 3:** pregnant teenagers´ perception of stigmatization

	ITEMS	SD 1	D 2	U 3	A 4	SA 5	MEAN
Personal stigmatisation	My friends are denied access to visiting me because I am pregnant	22	29	14	118	29	3.48
	My friends and neighbours jeer at/insult me when I go out	22	58	6	95	31	3.15
	I feel ashamed when I see neighbours and friends	19	63	7	93	30	3.25
	I am restricted from going out because I am pregnant	21	31	14	122	24	3.46
	I feel ashamed telling people about my pregnancy issues	16	78	15	72	31	4.47
	I would utilise MHS regularly if a special antenatal care is organised for pregnant teenagers	14	50	13	82	53	3.52
	The way people have treated me since I became pregnant upsets me	14	57	12	105	24	3.32
	Sub mean						3.52
Perceived stigmatisation	Other women laugh at me at the health centre	23	115	21	36	17	3.53
	Most people believe that pregnant teenagers are deviants	11	78	17	86	20	3.13
	Most people believe that pregnant teenagers should be punished for their pregnancy	13	75	12	88	24	3.17
	Sometimes I feel that I am being talked down to because I am pregnant	14	69	18	89	22	3.19
	Most teenagers would abort their pregnancy if they became pregnant	2	11	10	120	69	4.15
	Most people do not want to associate with pregnant teenagers	7	48	19	107	31	3.50
	Newspapers/television take a balanced view about teenage pregnancy	11	53	37	87	24	3.28
	Sub mean						3.42
	Grand Mean ± SD						3.4617

**Socio-cultural determinants of MHS utilisation among pregnant teenagers:** from Table 4, over 60% of respondents agreed that their parents allowed them to utilise MHS and on the contrary, about 61% disagreed that health care providers are hostile.

**Table 4:** determinants of MHS utilization

Determinants of MHS	Items	SD	D	U	A	SA	TOTAL
Permission from significant others	My parents do not allow me to attend ANC	53, 25%	11, 5.2%	20, 9.4%	118, 55.7%	10, 4.7%	212, 100%
	My partner does not allow me to attend ANC	52, 24.5%	13, 6.1%	21, 9.9%	119, 56.1%	7, 3.3%	212, 100%
	My mother-in-law does not allow me to attend ANC	52, 24.5%	119, 56.1%	26, 12.3%	9, 4.2%	6, 2.9%	212, 100%
	I will be punished for going against their decision	49, 23.1%	16, 7.5%	20, 9.4%	118, 55.7%	9, 4.2%	212, 100%
Attitude of health care providers	The health care providers are hostile	30, 25%	130, 5.2%	29, 9.4%	10, 4.7%	14, 7.2%	212, 100%
	The health care providers are judgmental	52, 14.2%	13, 61.3%	21, 13.7%	119, 56.1%	7, 3.3%	212, 100%
	The health care providers lack confidentiality	37, 17.5%	124, 58.5%	31, 14.6%	12, 5.7%	8, 3.8%	212, 100%
Religious beliefs	My religion does not support the use of orthodox medical services like MHS	51, 24.1%	114, 53.8%	15, 7.1%	18, 8.5%	14, 6.6%	212, 100%
	I believe that I will deliver safely without going to the hospital	17, 8%	69, 32.5%	17, 8%	84, 39.6%	25, 11.8%	212, 100%
	My religious head said I shouldn´t go to the hospital for any reason	56, 26.4%	119, 56.1%	23, 10.8%	8, 3.8%	6, 2.8%	212, 100%
Availability/ accessibility of MHS	There are few health care providers at the facility	13, 6.1%	63, 29.7%	36, 17%	84, 39.6%	16, 7.6%	212, 100%
	MHS facilities are not always open	24, 11.3%	74, 34.9%	34, 16%	67 31.6%	13, 6.1%	212, 100%
	MHS facilities are far from residential areas	20, 9.4%	45, 21.1%	33, 15.6%	93, 43.9%	21, 9.9%	212, 100%
	Patients have to wait for a long time to receive MHS	18, 8.5%	53, 25%	34, 16%	84, 39.6%	23, 10.9	212, 100%
Cultural beliefs	I feel safer using TBAs than MHS facility	29, 13.7%	92, 43.4%	30, 14.2%	40, 18.9%	21, 9.9%	212, 100%
	My culture restricts my use of maternal health services	41, 19.3%	118, 55.7%	26, 12.3%	13, 6.1%	14, 6.6%	212, 100%
Cost of MHS	It is very costly to use MHS	25, 11.8%	113, 53.3%	22, 10.4%	25, 11.8%	27, 12.7%	212, 100%
	I will not be attended to if I am not able to pay	18, 8.5%	45, 21.2%	23, 10.8%	93, 43.9%	33, 15.6%	212, 100%
	I will utilise MHS regularly if it is free	17, 8%	44, 20.8%	16, 7.5%	79, 37.3%	56, 26.4%	212, 100%
Coercion/violence from partner	My partner does not seek my opinion before taking decisions that regards my health	27, 12.7%	133, 62.7%	25, 11.8%	20, 9.4%	7, 3.3%	212, 100%
	My partner prohibits my use of MHS	43, 20.3%	132, 62.3%	20, 9.4%	10, 4.7%	7, 3.3%	212, 100%
	My partner threatens to hit me	42, 19.8%	133, 62.7%	17, 8%	13, 6.1%	7, 3.3%	212, 100%
	My partner hits me	41, 19.3%	139, 65.6%	16, 7.5%	3.8%	8, 3.8%	212, 100%

**Figure 1:**
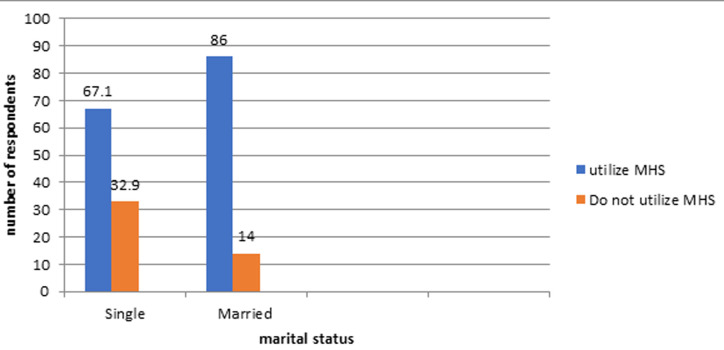
pattern of utilisation of MHS among married and unmarried pregnant teenagers

**Pattern of utilisation of MHS among married and unmarried teenagers:** Figure 1 reveals that, of the 155 pregnant teenagers that are single, 32.9% do not use MHS while 67.1% use MHS. Of the 57 pregnant teenagers that are married, 14% do not use MHS while 86% use MHS. This reveals that married pregnant teenagers use MHS more than their single counterparts.

**Association between maternal health service utilisation and maternal education, stigmatisation, availability and accessibility of MHS, cost of MHS and coercion and violence from partner:** Table 5, Table 6 and Table 7 show that there is a statistically significant association (Chi-square) between utilisation of MHS and maternal education (P=0.024), personal stigmatisation (P=0.001), perceived stigmatisation (P=0.014), availability and accessibility of MHS (P=0.002), cost of MHS (P=0.001) and coercion and violence from partner (P=0.000) among the respondents.

**Table 5:** chi-square analysis showing association between the utilisation of MHS and maternal education

Maternal education		Utilisation of MHS		Total	X^2^	Df	P-value	Remark
		No	Yes					
Level of education	None	6	6	12	5.8	3	0.024	Significant
	Primary	23	50	73				
	Secondary	28	83	111				
	Tertiary	2	14	16				
Total		59	153	212				
								
								
								

**Table 6:** chi-square analysis showing association of stigmatisation (personal and perceived) with the utilisation of maternal health care services

Personal Stigmatisation		MHS utilisation		X^2^	Df	P-value	Remark
		No	Yes	16.22	20	0.001	Significant
My friends are denied access to visiting me because I am pregnant	D	40	100				
	U	6	8				
	A	13	45				
My friends and neighbours jeer at/insult me when I go out	D	37	80				
	U	2	4				
	A	20	69				
I feel ashamed when I see neighbours and friends	D	39	73				
	U	3	4				
	A	17	76				
I am restricted from going out because of my pregnancy	D	40	103				
	U	4	10				
	A	15	40				
I feel ashamed telling people about my pregnancy issues	D	34	60				
	U	5	10				
	A	20	83				
I would utilise MHS regularly if a special antenatal care is organised for pregnant teenagers	D	23	41				
	U	6	7				
	A	30	105				
The way people have treated me since I became pregnant upsets me	D	37	82				
	U	4	8				
	A	18	63				
**Perceived stigmatization**				**14.31**	**20**	**0.014**	**Significant**
Other women laugh at me at the health center	D	34	104				
	U	13	8				
	A	12	41				
Most people believe that pregnant teenagers are deviants	D	30	59				
	U	5	12				
	A	24	82				
Most people believe that pregnant teenagers should be punished for their pregnancy	D	29	59				
	U	3	9				
	A	27	85				
Sometimes I feel that I am being talked down to because I am pregnant	D	30	53				
	U	6	12				
	A	23	88				
Most teenagers would abort their pregnancy if they became pregnant	D	3	10				
	U	3	7				
	A	53	136				
Most people do not want to associate with pregnant teenagers	D	25	30				
	U	7	12				
	A	27	111				
Newspapers/television take a balanced view about teenage pregnancy	D	28	36				
	U	15	22				
	A	16	95				

Key: Disagree ^*^D, Undecided ^*^U, Agree ^*^A

**Table 7:** chi-square analysis showing association between coercion/violence from partner, availability/accessibility of MHS, cost of MHS and the utilisation of MHS

Coercion/violence from		MHS utilisation						
Partner		No	Yes	Total	X^2^	Df	P-value	Remark
My partner does not seek my opinion before taking decisions that regards my health	D	34	126	160	25.077	8	0.001	Significant
	U	14	11	25				
	A	11	16	27				
My partner prohibits my use of MHS	D	38	137	175				
	U	11	9	20				
	A	9	7	17				
My partner threatens to hit me	D	43	132	175				
	U	8	9	17				
	A	8	12	20				
**Availability/accessibility of MHS**					**92.466**	**14**	**0.002**	**Significant**
MHS facilities are far from residential areas	D	3	62	65				
	U	21	12	33				
	A	35	79	114				
Patients have to wait for a long time to receive MHS	D	9	62	71				
	U	21	13	34				
	A	29	78	107				
There are few facilities that render MHS	D	9	83	92				
	U	20	16	36				
	A	30	53	83				
There are few health care providers at the facility	D	5	71	76				
	U	23	13	36				
	A	31	69	100				
MHS facilities are not always open	D	6	92	98				
	U	22	12	34				
	A	31	49	80				
**Cost of MHS**								
It is very costly to use MHS	D	27	111	138				
	U	14	8	22				
	A	18	34	52				
I will not be attended to if I am not able to pay	D	21	89	111	20.81	8	0.001	Significant
	U	15	8	23				
	A	22	56	78				
I will utilise MHS regularly if it is free	D	24	37	61				
	U	12	4	16				
	A	23	112	135				

In-depth interview:one of the respondents of the in-depth interview was 15 years while the others were between 18 to 19 years. Only two (2) were legally married, the rest were either single or co-habiting with their boyfriends/partners. Majority of them were Urhobo´s and understood pidgin English very well. They were all first-time pregnant teenagers. All of them were secondary school students and had to leave school when they became pregnant. Of the sixteen (16) pregnant teenagers interviewed, only four (4) utilised MHS from registered health facilities. One interviewee said: “I don´t go to the hospital. I go to that house over there (traditional birth attendant) to rub my stomach every week”. Another said: “I will deliver at home because my mother gave birth to all of us at home. My boyfriend also said that going to rub (TBA) is better than going to the hospital” (II3). When asked on their perception of MHS, one said: “MHS is good but my mother will not give me money for transport, she said I should get it from my boyfriend and he (boyfriend) doesn´t have money. My other friends that go to the hospital said they teach them many things that are good for the mother and baby” (II5). On stigmatisation, one 15-year-old first-time pregnant teenager who was a victim of rape emphasised stigmatisation as her main reason for not utilising MHS. She said: “everybody knows that I don´t know who made me pregnant and my parents are very angry, so I am always inside the house. I was drugged and raped at home; hence, I am always scared to go out because most people will laugh at me. Besides, everybody advised my parents to abort the baby but they refused” (II4). Another respondent added: “almost all my friends that became pregnant with me aborted it, so when they see that I am still pregnant they laugh at me, so I go out early in the morning to that greenhouse (TBA) to rub my stomach before other people see me” (II6).

All the respondents of the in-depth interview agreed that they would utilise MHS more if a special ANC was organised for them and wished it could be implemented immediately. One interviewee said: “Ah! (exclaims) That will be very good. If they make a special ANC, the whole place will be filled up because plenty people will come. Even all those people going to rub (TBA) will start coming to the hospital” (II3). On permission from significant others as a factor affecting utilisation of MHS, another interviewee had this to say: “I am new in this place, I travelled with my husband to this place and I don´t know anywhere here. Since I became pregnant seven (7) months ago, I have been inside the house, I don´t go anywhere because my husband would not allow me to go out. I told my husband to take me to the hospital, but he didn´t answer me” (II12). Another respondent also added that: “my mother said I shouldn´t go to the hospital that I would deliver at that woman´s house [TBA] because if I go to the hospital, they would use operation (caesarean section) to bring out the baby”. On the accessibility of MHS as a factor affecting utilisation of MHS, an interviewee responded saying: “I like to go to the hospital but the one we have here is always closed. Most people go to the health centre in the other village and it is very far. I have only gone there once when I was four (4) months pregnant and since then I haven´t gone again because it´s too far and I don´t have money for transport” (II2). Also, another respondent revealed that: “it will be good if they can make the hospitals closer to our house and if they can attend to us on time so that I can quickly use their services and return home before my boyfriend comes back” (II9). On the cost of MHS as a major determinant of MHS utilisation, one of the interviewees said: “I love to go to the hospital (ANC) to assess the state of my pregnancy but I don´t have money. When I asked my mother for money, she referred me to my boyfriend who also doesn´t have money” (II4). Another respondent who was a school dropout now a petty trader said: “I am saving my money so that when it is time to deliver, I will go the hospital to have my baby delivered. I don´t have enough money to go for ANC; I would only deliver there”. Yet another added that: “They [nurses and health care workers] should try and attend to us even when we don´t have money because they don´t attend to some people that do not come with money” (II6).

On coercion and violence as a factor affecting utilisation of MHS, one of the two interviewees responded thus: “my boyfriend [whom she co-habits with] always hits me whenever we have a disagreement and I suffer severe pains for weeks. This makes me unable to go out and he also doesn´t give me money for ANC” (II2). Another interviewee said: “My boyfriend [whom she co-habits with] always hits me for no reason. This is because he requested that I terminate the pregnancy which I objected to. Hence, I can´t ask him for money. I only rely on gifts from friends which I am currently saving to procure baby items when I eventually put to bed" (II3).

## Discussion

**Statement of principal findings:** the socio-demographic characteristics of the pregnant teenagers reveal that majority of the pregnant teenagers were between 18 to 19 years (63.7%). This shows that pregnancy rate among the very young teenagers (13 to 15 years) is on the decrease in Southern Nigeria as opposed to what is obtainable in the Northern part of the country. Also, on educational attainment, only 40.1% of these teenagers were students. This shows that a good number of female children do not still have access to education as supported by the findings that 34 million girls worldwide are absent from secondary school [18]. This calls for a need to increase awareness on the importance of the girl child education as failure to educate the girl child would decrease the country´s productivity in the long run. On marital status, 73.1% of the study participants were single while 26.9% of them were married. Only 10% of the 73.1% that were single utilise MHS, thus implying that single pregnant teenagers are less likely to utilise MHS than their married counterparts. Similar findings have also been reported that married women were more likely to go for ANC and make their first visit during the first trimester compared to their never-married counterparts [19]. Also, it has been documented that single mothers and pregnant women who are not in a stable relationship were less likely to attend ANC than married women [20]. Encouraging teenagers to attain independence and have a stable relationship will help increase their MHS utilisation when and if they eventually become pregnant.

**Extent of utilisation of MHS:** results of the study further revealed that 72.2% of the participants claimed to utilise MHS by visiting an ANC centre at least once during their pregnancy which is in line with the findings that only about three-fifths (60.3%) of women of childbearing age used antenatal services at least once during their most recent pregnancy [21]. Of this 72.2%, only 60 (28.3%) participants in total had ANC attendance that was appropriate with their gestational age. A 21% (low level of utilisation of MHS) extent of MHS use by pregnant teenagers has been documented [17]. However, when compared with women of childbearing age, a 57% utilisation rate has been reported in the same Delta State [22]. This further emphasises the already established fact that pregnant teenagers have a lower rate of MHS utilisation than older women despite their health and obstetric risks.

**Determinants of MHS:** several socio-cultural factors were revealed to be associated with the utilisation of MHS. Permission from significant others was one of these factors. This study has shown that most pregnant teenagers need permission from their spouse, mother-in-law or mother to utilise MHS and some even get punished when they try to go against their decision. This is in line with the position of [23] who identified husband´s permission to use health services among several other socio-cultural factors as barriers to women´s use of hospital delivery (a component of MHS). This is further corroborated by [24] who documented that ANC usage of 54.2% was found among women who needed their husband´s permission to seek ANC compared with 67.4% among those who did not need it. The impact of stigmatisation (personal and perceived) and negative stereotypes on the utilisation of MHS was also greatly stressed by the majority of the study participants. This finding is in line with [25] who reported that stigma should be of concern to health providers. Stigmatising practices hampers effective health care, contribute to teen mothers´ many challenges and violate the nursing ethic that patients be treated with respect and dignity. Timely interventions to curb these identified factors would help increase the rate of utilisation of MHS by pregnant teenagers. Availability/accessibility of MHS is another socio-cultural determinant of MHS utilisation in this study. Most of the respondents implicated insufficient MHS centres as a reason for non-utilisation of MHS and where available, are situated far from residential areas. This is in line with the position of [26] that adolescents who live in neighbourhoods with an antenatal care clinic were more likely to begin receiving care earlier in pregnancy. This is further corroborated by the findings of [27] who reported that the decision to deliver in a health facility is also associated with proximity to the facility, cost and quality of care.

The recommendations by [28,29] on the need to establish more ANC rendering facilities in rural areas remain valid to improve MHS utilisation. Also, cost of MHS is another crucial factor that was found to determine the utilisation of maternal health care services by pregnant teenagers. This is in agreement with the position of [24] who documented that financial hindrances were cited by about two-fifths of women not attending ANC services in Nigeria and that with the high level of poverty in the country, financial cost serves as a barrier to the use of ANC services by some women, particularly the most vulnerable (the poor and young mothers). Also, [30] documented that 65.3% of their study participants (women of childbearing age) claimed that affordability of services played a key role in their choice of health care utilisation. However, it was generally agreed upon by all the pregnant teenagers in this study that a special ANC for pregnant teenagers with little or no cost implication would greatly help to scale up their utilisation of MHS. Factors like religion and culture were not implicated as determinants for MHS utilisation in this study. However, the impact of religion and culture in determining MHS utilisation among pregnant teenagers lies in the fact that it plays a significant role in shaping beliefs, norms and values including those that relate to childbirth and health service use [23].

**Recommendations:** the study has been able to reveal the extent of utilisation of maternal health care services in Delta State, Nigeria. While it has been found that a good number of pregnant teenagers utilise maternal health care services by visiting an ANC centre at least once during their pregnancy, a lot needs to be put in place to ensure adequate compliance and utilisation of MHS. These include: financial empowerment of these pregnant teenagers or a free-of-charge ANC would help to scale up its utilisation; pregnant teenagers who are victims of intimate partner violence (IPV) should be encouraged to speak up as well as channels through which their voices can be heard should be created; awareness on the danger of early marriage as well as unlawful cohabitation is necessary to decrease the incidence of IPV among pregnant teenagers; the number of health workers, especially midwives and Community Health Extension Workers [CHEWS], need to be increased so that special and individualised care can be given to pregnant teenagers; this study also has an implication for training of the workers in the ANC centres on the need for culturally competent care as some of the pregnant teenagers reported that nurses sometimes gave an unreceptive attitude when they turn up for ANC visits. This is a problem that needs to be addressed; finally, family-centred care approach should be employed in the provision of MHS to pregnant teenagers as the decision to utilise MHS greatly lies on the significant others (spouse, parents and mother-in-law) of the pregnant teenager.

## Conclusion

The utilisation of MHS has been proven to be very effective in the reduction of maternal mortality and morbidity among pregnant teenagers and childbearing women at large. While teenage pregnancy is on the decrease in developed countries, developing countries like Nigeria still has a high percentage of pregnant teenagers. This study has provided a baseline statistic of the extent of utilisation of maternal health services and its determinants by pregnant teenagers in Delta State, Nigeria. Time has come to focus on this group of vulnerable adolescents and their utilisation of MHS since efforts to curb the ugly trend of teenage pregnancy has failed especially in developing countries where our cultural inclinations primarily regulate our actions.

### What is known about this topic

- Use of maternal health care services is a key proximate determinant of maternal and infant outcomes, including maternal and infant mortality for pregnant teenagers;
- Teenagers have increased risk for poor maternal and infant outcomes and it is widely assumed that they are less likely than older women to use maternal health services;
- Utilisation of maternal health care services is influenced by cultural, religious, political, economic and social factors.

### What this study adds

- Only 28.3% of pregnant teenagers utilise MHS and have ANC attendance that is appropriate with their gestational age in Delta State, Nigeria;
- Availability of health personnel, accessibility to MHS facilities, permission from significant others and cost of MHS are determinants of MHS utilisation in Delta State, Nigeria;
- Married teenagers (86%) were found to utilise MHS more than their single counterparts (67.1%) in Delta State, Nigeria.
